# Detection of indicator polychlorinated biphenyls (I-PCBs) and polycyclic aromatic hydrocarbons (PAHs) in cow milk from selected areas of Dhaka, Bangladesh and potential human health risks assessment

**DOI:** 10.1016/j.toxrep.2022.07.008

**Published:** 2022-07-12

**Authors:** G. M. M. Anwarul Hasan, Md. Aftab Ali Shaikh, Mohammed A. Satter, Md. Sabir Hossain

**Affiliations:** a Institute of Food Science and Technology (IFST), Bangladesh Council of Scientific and Industrial Research (BCSIR), Dr. Qudrat-E-Khuda Road, Dhaka 1205, Bangladesh; b Department of Chemistry, University of Dhaka, Dhaka 1000, Bangladesh; c Bangladesh Council of Scientific and Industrial Research (BCSIR), Dhanmondi, Dhaka 1205, Bangladesh; d Department of Biochemistry & Molecular Biology, Jahangirnagar University, Savar, Dhaka 1342, Bangladesh

**Keywords:** Pesticides, Hexachlorocyclohexane, Heavy metals, Health hazards, POPs, GC-MS/MS

## Abstract

In this study, the levels of indicator PCBs congeners and PAHs compounds were reported in cow milk from selected areas in Dhaka, Bangladesh, and the potential human health risks were assessed. A total of 100 milk samples were collected and analyzed using gas chromatography-tandem mass spectrometry (GC-MS/MS). Method validation was performed using recovery performance, linearity, limit of detection (LOD), and limit of quantification (LOQ) assays. PCBs congeners, including PCB No. 52 (2,2´,5,5´-tetrachlorobiphenyl), PCB No. 101 (2,2´,4,5,5´-pentachlorobiphenyl), PCB No. 153 (2,2,4,4,5,5–hexachlorobiphenyl), and PCB No 209 (Decachloro-1,1′-biphenyl perchlorobiphenyl) were detected, whereas PCB No 28 (2,2 ´,4–trichlorobiphenyl), PCB No. 138 (2,2´,3,4,4´,5´–hexachlorobiphenyl), and PCB No 180 (2,2´,3,4,4´,5,5´–heptachlorobiphenyl) were not detected in the analyzed milk samples. Among the 16 PAHs compounds, benzo (a) anthracene and chrysene were detected in milk samples. The Σ hazard risk index (HI) of all detected PCBs congeners was below the limit set by the European Food Safety Authority, which indicates limited health risks to animals and humans in the study area. However, the presence of PCBs and PAHs in milk samples from industrial areas may negatively affect human health, and further detailed studies are required to ensure public health safety.

## Graphical Abstract

**Figure:**
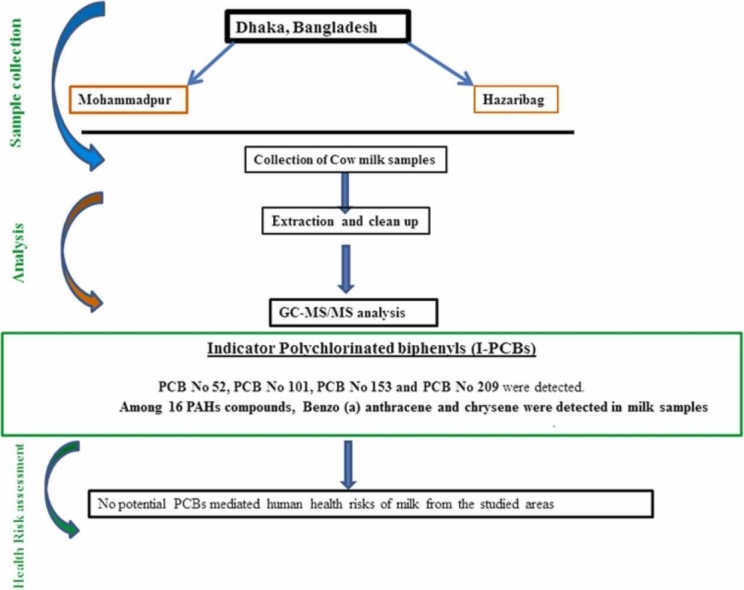

## 1 Introduction

Biogenic and anthropogenic sources introduce pollutants into the environment. However, human activities are primarily responsible for their massive discharge into the atmosphere. Polychlorinated biphenyls (PCBs), hexachlorocyclohexane (HCH), polybrominated diphenyl ethers (PBDEs), polyaromatic hydrocarbons (PAHs), pesticides, and heavy metals are anthropogenic inorganic and organic pollutants that are dispersed throughout the atmosphere, hydrosphere, and lithosphere. They may be transformed into other compounds that are even more toxic to flora and fauna [1]. Because of their industrial uses, several contaminants, including PCBs and PAHs, are mixed with the environment, which is very hazardous and is retained in the environment, posing a severe threat to both public health and the ecosystem [2], [3], [4], [5], [6], [7], [8], [9].

PCBs are long-lasting contaminants that result from the defective combustion of organic and synthetic materials [10]. PCBs are synthetic organic compounds found in most industrial and consumer items. The production of PCBs was banned in the USA in 1977 [11], [12]. Because of their persistence and bioaccumulation, PCBs are categorized as persistent organic pollutants (POPs) [13]. The major concern is the presence of PCBs in consumer items such as plasticizers in paints, plastics, and rubber products. As PCBs accumulate in adipose tissue, they are typically found in animal-derived foods. PCB exposure is commonly caused by eating fish; however, other foods with lower PCB levels that are consumed more frequently, such as beef, dairy, and chicken products, also contribute to PCB exposure [11]. PCBs are causative agents of cancer, immune system diseases, and malfunctions in the reproductive, neurological, and other biological systems [14]. PCBs are widely used as pollutant markers because of their presence in biotic and abiotic conditions [15].

PAHs comprise hydrogen and carbon fused with two or more aromatic rings [16]. The characteristics of PAHs include a high melting point, low vapor pressure, and low aqueous solubility [17]. They are ubiquitous pollutants that are formed during food processing [18]. Normally, PAHs consisting of five or more rings are potentially hazardous to humans, which is a great concern for public health [19], [20], [21]. PAHs can be categorized based on the presence of aromatic rings [22]. LMW PAHs show acute toxicity, whereas HMW PAHs show high carcinogenicity [23]. PAHs are generally considered to be organic pollutants [22]. Because of the presence of PAHs in food, their control is necessary for public health safety [24]. Most PAHs are exposed to the human body through diet in different countries [19], [20], [21]. The major problem is that PAHs are not normally detected in raw foods [25].

PAHs can contaminate foods from environmental pollution or during food preparation and processing. Most gas-phase PAHs compounds are adsorbed in the particulate matter and finally accumulate in water and soil with heavy PAHs, which enter the food chain through vegetation and plant foods. Therefore, grazing cows can uptake PAHs from the soil through their feed and accumulate them in animal-origin foods [26]. In food, PAHs are present as a complex mixture of light and heavy compounds [27]. The other possible ways of accumulating PAHs through food consumption are smoking, drying, and fuel combustion [28]. Foods processed at high temperatures can also generate PAHs. According to one study, PAHs exposure in non-smokers occurs through food consumption [29]. PAHs have harmful effects on human health through carcinogenesis, mutagenesis, and immunosuppression. Several studies in animals and humans have demonstrated that PAH exposure is associated with increased cancer prevalence [28], [29], [30].

Several reports have demonstrated that products with high-fat content are important sources of human exposure to recognized toxins in the environment. Consequently, fatty foods, including meat, milk, and eggs, may contain significant amounts of POPs and other chemicals [30]. Harmful products, such as PCBs, can be consumed through the human diet. However, the principal sources of human exposure to lipophilic PCBs are fatty meals of animal origin [31], [32]. After discharge into the environment, these organic pollutants may accumulate in the soil and grasses. Milk and milk products are important sources of the human diet. Milk products also can be contaminated through PAHs. Therefore, a proper food management system should be established to ensure public health safety. PAHs are lipophilic and can accumulate in fats, particularly in foods of animal origin. Milk and dairy products are important sources of PAHs because milk can be contaminated by ingesting contaminated feed, grass from contaminated soil, and soil [33], [34], [35].

The impacts of PCBs and PAHs, PCBs and PBDEs are considered seriously because of their persistence nature in the environment [36]. Milk containing environmental pollutants is a major health threat. Therefore, monitoring milk samples is necessary to assess potential health risks. PCBs are used in different products, and PCB-containing waste is stored in landfills. It has been estimated that approximately 10% of the PCBs produced earlier still exist in the environment. Several by-products can be formed from PCBs through burning, which can be easily dissolved in water rather than evaporating. PCBs are fat-soluble and stored in animal fat through the food chain. Humans are mainly exposed to fat-rich foods. People can also have a small amount of PCBs through breathing contaminated air. PCBs have several effects on human health, including fertility, child growth, the immune system, and cancer development. The United States Environmental Protection Agency (USEPA) has identified 16 PAHs compounds as ‘priority pollutants and has been monitored extensively [37], [38]. The European Union (EU) listed 15 PAHs compounds in monitoring studies in 2005 [37]. Several compounds, including benzo (a) anthracene, chrysene, benzo (b) fluoranthene, benzo (k) fluoranthene, benzo (a) pyrene, indeno (1,2,3-cd)pyrene, benzo (ghi) pyrene, and dibenzo (ah) were identified as highly potential carcinogenic compounds compared to others [38]. PAHs such as BaA, Chr, BbF, and BaP are classified as mutagenic, genotoxic, and carcinogenic compounds by The International Agency for Research on Cancer (IARC) and Joint Food and Agriculture Organization/World Health Organization (FAO/WHO), Scientific Committee on Food (SCF), and European Food Safety authority (EFSA).

Although the presence of PCBs and PAHs in fat-rich foods, such as milk, is a major health concern, not much work has been done so far in Bangladesh. This study aimed to determine the levels of PCBs and PAHs in cow milk from selected areas in Bangladesh and assess possible human health risks.

## 2 Material and methods

### 2.1 Area of study

A total of 100 raw milk samples from 10 sampling sites were collected from the dairy firms of Mohammadpur and Hazaribag in Dhaka. The samples were collected between October 2020 and February 2021. Hazaribag is used in the leather industry. The site selection was based on industrial and agricultural activities. Many leather and tanning industries are located in Hazaribag. Mohammadpur is adjacent to the Turag River. The water of the Turag River is contaminated by industrial waste. Moreover, several waste dumping sites are located near both sampling areas, where the wastes are burned regularly. Milk samples were collected from dairy farms and local shops. Raw milk samples were used in this analysis. About 500 mL of milk samples were collected in plastic polythene bags, transported to the laboratory on ice box and stored at 4ºC and analyzed within 24 h of sample collection.

### 2.2 Chemicals and standards

A cocktail standard mix of PCBs congeners including PCB No 28, PCB No 52, PCB No 101, PCB No. 138, PCB No. 153, PCB No.180, and PCB No. 209 (PCB standard solution 7) and a cocktail mix of 16 PAHs compounds containing Napthalene, 2-methylnapthalene, 1-methylnapthalene, Acenapthylene, Acenapthalene, Fluorene, Phenanthrene, Anthracene, Fluranthene, Pyrene, Benzo (A) Anthacene, Chrysene, Benzo (B) Fluoranthene, Benzo (K) Fluranthene, Benzo (A) Pyrene, Indeno (1, 2, 3-CD) Pyrene and Dibenz (A, H) Anthracene brought from Sigma Aldrich (Darmstadt, Germany). Acetonitrile (Advent Chembio, India) and other chemicals including n-hexane, magnesium sulfate, sodium acetate, and NaCl were purchased from Merck (Darmstadt, Germany).

### 2.3 Extraction and cleanup

Sample extraction was performed using the quick, easy, cheap, effective, rugged, and safe (QuEChERS) method [39], [40], [41] with slight modifications for PCBs analysis. Samples were brought to normal temperature, and 5 mL of milk was transferred to a 50 mL polypropylene tube. After dilution with ultrapure water, the samples were extracted with 10 mL hexane: acetone (1:1, v/v). Anhydrous magnesium sulfate (4 g) and sodium chloride (1 g) were added to the mixture and shaken for 5 min at 175 rpm. Thereafter, the mixture was centrifuged at 3500 rpm for 5 min. Approximately 10 mL of the supernatant was poured into a glass tube, and the solution was dried using a nitrogen stream at 40ºC the solution was dried. Subsequently, the samples were cleaned, and the eluates were evaporated using a nitrogen stream at 40 °C. Finally, the extracts were dissolved in 100 µL of isooctane for GC-MS/MS analysis.

For PAHs analysis, samples were first homogenized, and 5 g of homogenized samples were placed in a conical tube and shaken for 1 min. A mixture of hexane and acetonitrile (1:1 v/v) was prepared, 15 mL of the mixture was added, and the mixture was shaken for a few seconds. Subsequently, magnesium sulfate (6 g) and sodium acetate (1.5 g) were added to the mix [42]. The solubility of the PAHs was reduced by adding NaCl to the mixture. The mixture was then centrifuged at 2500 g for 5 min. The supernatant was removed, and PAHs were isolated through solid-phase extraction (SPE). Subsequently, activated silica gel and 1 g of Na_2_SO4 were loaded onto a glass chromatographic column, and n-hexane was used. The concentrated extracts were dissolved in 5 mL of n-hexane, loaded into the column, and eluted with 50 mL of n-hexane. Finally, the eluents were concentrated and dissolved in n-hexane (0.5 mL of n-hexane for GC-MS/MS analysis [43].

### 2.4 Analysis of PCBs congeners and PAHs compounds in milk samples

PCBs congeners were analyzed using a gas chromatograph coupled with a MS (Thermo Scientific, USA). Analytical separations were performed using a Trace GOLD™ TG-5MS GC Column (0.25 mm × 0.25 µm X 0.25 m). Helium was used at a constant pressure (18.29 psi) at 1.6 mL/min flow rate. The injection port temperature was 250 °C, and the initial oven temperature was 60 °C, which was further increased to 220ºC at 5ºC/min heating ramp. The detector temperature was approximately 320 °C during the sample analysis. The sample injection volume was 2 µL.

For the PAHs analysis, helium was used as the carrier gas at 1.8 mL/min flow rate. The injection volume was 1 µL in split-less mode. The ion source and quadrupole temperatures were 280 °C and 180 °C, respectively, whereas the injector and transfer line temperatures were 280 °C and 310 °C, respectively. The column temperature was initially set at 35 °C for 1 min, then increased to 200 °C at a ramp rate of 25 °C/min, and finally ramped to 310 °C at 8 °C/min and kept for 3.5 min. Both of PCBs and PAHs analyte detection was carried out using MS at 70 eV ionization energy in selected ion monitoring (SIM) acquisition mode.

### 2.5 Method quality control

Working solutions were prepared by diluting standard stock solutions with n-hexane. For the linearity test, working solutions of several dilutions in the range of 50–200 µg/L were prepared and injected into the GC-MS/MS system. The LOQ and LOD were determined using the signal-to-noise ratio. Method validation was performed using a recovery performance evaluation. The recovery percentage was determined using the following equation:

(1) Pi= (Si/Ti) × 100Pi= (Si/Ti) × 100

Here, Pi is the recovery percentage, Si is the result of the control, and Ti is the percent recovery of the spiked samples.

### 2.6 Health risk assessment of PCBs congeners

Human exposure to chemicals in food is estimated using three different parameters: substance concentration in food (ng/kg lipid weight (l.w)), food consumption rate (kg), and population body weight (kg).

The estimated daily intake (EDI) is determined through the following equation:

(2) EDI = (DFC×FCC)/BW

In this case, EDI is the estimated daily intake (g day−1), DFC indicates daily food consumption (kg/day), FFC indicates the food chemical concentration present in the analyzed food (mg/kg), and BW indicates the body weight (kg) of consumers. In this analysis, an average body weight of 70 kg/per adult person was considered for calculating the dietary exposure. The per capita milk consumption per day in the Bangladeshi population is 27.31 g [44].

### 2.7 Hazard Risk Index (HI) of PCBs congeners

The following formula was used for HI calculation:

(3) HI = EDI/ADI

EDI: estimated daily intake of PCBs; ADI: acceptable daily intake through milk consumption. HI > 1 is considered a potential health hazard, and HI< 1 is considered safe for public health [45], [46].

### 2.8 Statistical analysis

Data were processed using mass spectrometry. The peak areas were considered for PCB quantification. Analytical data were collected and organized using Microsoft Excel. Mean concentration differences and standard deviations in milk samples, in accordance with the sampling sites, were determined using one-way ANOVA. Samples below the LOD values were considered to have a concentration of 0.0 for statistical analysis. Median, Interquartile range of detected analytes were calculated using Calculator Soup online software.

## 3 Results and discussion

### 3.1 Method performance

**Fig. 1:**
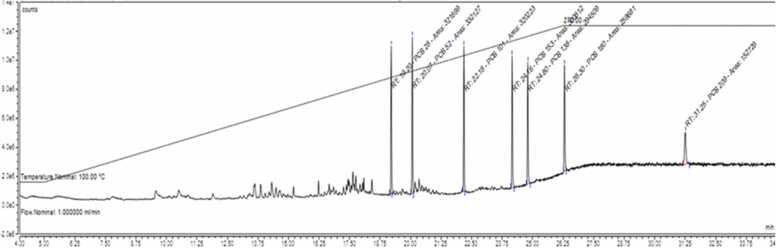
Chromatogram showing the peaks of PCBs congeners in standard solution. The elution order is as follows: 1: PCB 28; 2: PCB 52; 3: PCB 101; 4: PCB 153; 5: PCB 138; 6: PCB 180 and 7: PCB 209.

**Fig. 2:**
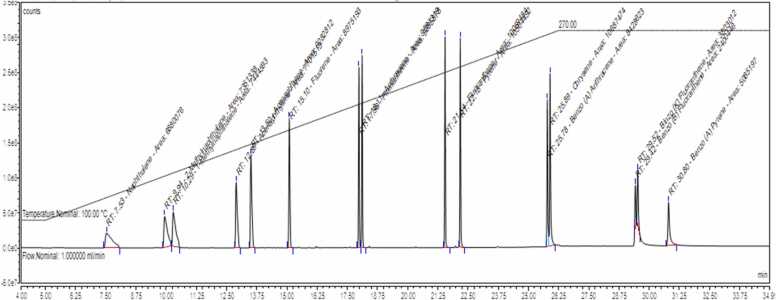
Chromatogram showing the peaks of PAHs compounds in standard solution.

There are two common methods for the detection of PCBs in food samples: GC with an ionizing flame detector and MS [37], [47], and liquid chromatography with a fluorescence detector [48], [49], [50] are used. There are several methods for the detection of PAHs in milk products, including HPLC-FLD [51], [52], high-performance liquid chromatography–ultraviolet detection (HPLC-UV) [53], [54], [55], [56], [57] and GC-MS [35], [58], [59] or GC-MS/MS [24]. Because of its sensitivity and accuracy, we used GC-MS/MS in this study to determine PCBs and PAHs in cow milk. The chromatograms of the residual peaks of PCBs and PAHs are shown in Figs. 1 and 2, respectively. The method was validated based on recovery performance. Blank samples were adulterated with 50 ng/g and 100 ng/g PCBs congeners and PAHs compounds, respectively. Later, the adulterated samples were compared with those of the blank samples. In this study, the recovery rate of the PCBs were varied from 77.53% to 92.49%, while LOD and LOQ values were detected in the range of 0.016 ng/g to 0.031 ng/g l.w and 0.059 ng/g to 0.08 ng/g l.w, respectively. Table 1 lists the LOD, LOQ values, and % recoveries of each PCBs. In contrast, PAHs compounds were recovered in the range of 67.90–99.76%. The LOD and LOQ values were 0.3, 1.0, and 1.0, 4.0 respectively. The elution order, LOD, LOQ, and recovery rate of PAHs are shown in Table 2.

**Table 1:** The percent recoveries, LOQ and LOD values of PCBs congeners.

PCBs	**50 ng/ g**	**100 ng/ g**	**LOQ**	**LOD**
PCB No 28	92.49 ± 5.73	81.49 ± 5.69	0.069	0.019
PCB No 52	88.33 ± 6.42	88.32 ± 4.65	0.07	0.027
PCB No 101	87.82 ± 4.86	90.21 ± 6.97	0.074	0.031
PCB No. 138	92.35 ± 5.21	82.63 ± 4.04	0.061	0.019
PCB No. 153	82.43 ± 6.33	77.53 ± 5.54	0.059	0.016
PCB No 180	80.54 ± 4.98	83.23 ± 7.42	0.066	0.022
PCB No 209	89.23 ± 5.75	83.33 ± 5.95	0.074	0.028

**Table 2:** Elution order, limit of detection, limit of quantification and recovery rate of 16 PAHs compounds.

**Analyte**	**Elution Order**	**LOQ**	**LOD**	**% recovery**	
		(ng/g)	(ng/g)	50 ng/g	100 ng/g
Napthalene	1	1	0.4	74.43 ± 4.48	67.98 ± 6.54
2-methylnapthalene	2	1	0.3	76.9 ± 7.56	73.89 ± 9.65
1-methylnapthalene	3	1	0.4	68.54 + 9.54	69.09 ± 5.78
Acenapthylene	4	1	0.4	75.09 ± 4.49	76.87 ± 9.56
Acenapthalene	5	1	0.3	70.56 ± 5.67	83.89 ± 8.85
Fluorene	6	1	0.3	68.98 ± 7.02	80.67 ± 9.07
Phenanthrene	7	2	0.5	70.78 ± 0.76	99.90 ± 7.54
Anthracene	8	2	0.5	95.98 ± 9.56	90.65 ± 6.67
Fluranthene	9	2	0.5	88.23 ± 3.89	85.76 ± 4.58
Pyrene	10	2	0.5	81.09 ± 8.98	80.65 ± 4.47
Benzo (A) Anthacene	11	3	0.9	76.67 ± 6.87	69.67 ± 7.98
Chrysene	12	3	0.8	67.90 ± 6.65	92.89 ± 7.67
Benzo (B) Fluoranthene	13	3	0.9	74.98 ± 7.89	76.98 ± 4.55
Benzo (K) Fluranthene	14	3	0.8	88.98 ± 9.09	66.89 ± 6.54
Benzo (A) Pyrene	15	3	0.8	92.87 ± 7.45	90.87 ± 5.66
Indeno (1, 2, 3-CD) Pyrene	16	4	0.9	99.76 ± 6.74	74.56 ± 9.77
Dibenz (A, H) Anthracene	17	4	0.8	92.90 ± 8.43	86.54 ± 5.90

### 3.2 Detection of PCB residues in milk

The levels of PCBs in cow milk samples collected from local firms in Dhaka, Bangladesh, were determined using GC-MS/MS. In this study, 100 milk samples were collected. PCBs residues including PCB No 52 (0.63 ± 0.21 ng/g l.w), PCB No 101 (0.52 ± 0.22 ng/g l.w), PCB No 153 (4.75 ± 0.41 ng/g l.w), and PCB No 202 (5.35 ± 0.87 ng/g l.w) were detected, whereas PCB No 28, PCB No 138, and PCB No 180 were not detected or detected below the detection limit (<LOD). The mean values, median, Interquartile range of detected PCBs congeners and Percentage of Positive samples are shown in Table 3, Table 4 respectively. Among the detected PCBs residues, PCB 209 was detected at the highest concentrations, followed by PCB 153, PCB 52, and PCB 101. The First quartile, second quartile, third quartile and Interquartile Range values of PCB 52 were 0.49, 0.59, 0.76 and 0.27 respectively. For PCB 101, the values were 0.415, 0.485, 0.615 and 0.2 respectively. The first quartile, second quartile, third quartile and Interquartile Range values of PCB 153 were 2.13, 2.87, 3.25 and 1.12 respectively. For PCB 209, the values were 2.945, 3.45, 3.71 and 0.765 respectively.

**Table 3:** Mean concentration ± SD of detected PCBs in cow milk samples of Dhaka, Bangladesh.

**PCBs**	**Mean Concentration (ng/kg l.w)**
PCB No 28	ND
PCB No 52	0.63 ± 0.21
PCB No 101	0.52 ± 0.22
PCB No. 138	ND
PCB No. 153	2.75 ± 0.41
PCB No 180	ND
PCB No 209	3.35 ± 0.87

* ND: Not detected

**Table 4:** Median, Interquartile range of detected PCBs congeners and Percentage of Positive samples.

**PCB No 52**	**% of Positive samples**	**PCB No 101**	**% of Positive samples**	**PCB No. 153**	**% of Positive samples**	**PCB No 209**	**% of Positive samples**
First Quartile	70	First Quartile	80	First Quartile	70	First Quartile	50
0.49		0.415		2.13		2.945	
Second Quartile		Second Quartile		Second Quartile		Second Quartile	
0.59		0.485		2.87		3.45	
Third Quartile		Third Quartile		Third Quartile		Third Quartile	
0.76		0.615		3.25		3.71	
Interquartile Range		Interquartile Range		Interquartile Range		Interquartile Range	
0.27		0.2		1.12		0.765	
Median = Q2		Median = Q2		Median = Q2		Median = Q2	
0.59		0.485		2.87		3.45	
Minimum		Minimum		Minimum		Minimum	
0.46		0.39		1.78		2.68	
Maximum		Maximum		Maximum		Maximum	
0.97		0.77		3.68		3.76	
Range		Range		Range		Range	
0.51		0.38		1.9		1.08	

### 3.3 Determination of PAHs compounds in milk

The mean values, median, interquartile range of detected PAHs analytes and Percentage of Positive samples of PAHs in the milk samples are presented in Table 5, Table 6 respectively. Among the 16 PAHs compounds, only benzo (a) anthracene and chrysene were detected. The concentrations of Benzo(a) anthracene and chrysene were 0.5497 ± 0.30 ng/g and 1.077 ± 0.878831 ng/g, respectively. The First quartile, second quartile, third quartile and Interquartile Range values of Benzo (A) Anthacene were 0.34, 0.435, 0.75 and 0.41 respectively. For Chrysene, the values were 0.465, 0.68, 1.275 and 0.81 respectively. Contaminated samples were detected from 2 of the 10 sampling sites. Samples collected from nearby industrial areas contained these two PAHs compounds, whereas samples from other sampling sites did not contain any PAHs compounds. The concentrations of other compounds were not detected in any samples. The absence of those compounds indicated low contamination rate in the studied area.

**Table 5:** Detected PAHs compounds (ng/g) in Cow milk.

	**Mean (ng/g)**
Napthalene	ND
2-methylnapthalene	ND
1-methylnapthalene	ND
Acenapthylene	ND
Acenapthalene	ND
Fluorene	ND
Phenanthrene	ND
Anthracene	ND
Fluranthene	ND
Pyrene	ND
Benzo (A) Anthacene	0.5497 ± 0.304514
Chrysene	1.077 ± 0.878831
Benzo (B) Fluoranthene	ND
Benzo (K) Fluranthene	ND
Benzo (A) Pyrene	ND
Indeno (1, 2, 3-CD) Pyrene	ND
Dibenz (A, H) Anthracene	ND

* ND: Not detected

**Table 6:** Median, Interquartile range of detected PAHs analytes and Percentage of Positive samples.

**Benzo (A) Anthacene**		**Chrysene**	
First Quartile	% of Positive samples	First Quartile	% of Positive samples
0.34	80	0.465	90
Second Quartile		Second Quartile	
0.435		0.68	
Third Quartile		Third Quartile	
0.75		1.275	
Interquartile Range		Interquartile Range	
0.41		0.81	
Median = Q2		Median = Q2	
0.435		0.68	
Minimum		Minimum	
0.19		0.38	
Maximum		Maximum	
1.12		3.98	
Range		Range	
0.93		3.6	

### 3.4 Health risk from the consumption milk from the studied area

The EDI and HI values of the PCBs are presented in Table 7. The HI values of PCBs No. 52, No. 101, 153, and 209 were 5.58 × 10^−6^, 4.61 × 10^−6^, 4.20 × 10^−5^, and 4.73 × 10^−5^, respectively. From the risk analysis, it can be concluded that the milk samples from the study area were safe for consumers, as the HI values were far below the risk level. However, milk samples containing PCBs residues for a longer time may cause health hazards. The results obtained from the method validation were satisfactory for the analysis of PCBs and PAHs. Table 3 shows the mean concentrations (ng/g l.w) of PCBs detected in milk samples. Among the 100 analyzed milk samples, PCB No. 52, 101, 153, and 209 were detected, whereas some PCBs, including PCB No. 28, 138, and PCB No. 180 showed values below the limit of detection. The PCBs concentrations detected in this study were lower than those reported in other studies [60], [61], [62]. Findings from Japan have indicated that intake of PCB-contaminated foods is toxic to mothers [63]. Milk processing at higher temperatures could lower PCBs concentrations, as it has been reported in several studies that pesticide concentration is reduced after thermal processing [64], [65], [66]. According to the regulation of 1259/2011, the Committee for European Communities has established a PCB reference limit of 40 ng/g fat [67] According to the regulation of 1259/2011, the committee for European communities has established a reference limit of PCBs of 40 ng/g fat [65]. As there have no established Maximum Residue Limits (MRLs) values for PCBs concentrations in cow milk in Bangladesh, therefore, no comparisons in PCBs in milk can be done in Bangladeshi perspective. Animal-derived foods (both terrestrial and aquatic) are significant components of the human diet because they supply essential minerals [68], [69], [70] for human health. Various researchers throughout the world have detected the presence of PAHs and PCBs in protein source foodstuffs [28], [71], [72], [73]. Higher concentration of chrysene (12.56 ± 19.17 ng/g) and small quantity of benz(a)anthracene (0.30 ± 0.46 ng/g) was detected in cow milk of southern Italy [74] while in Egypt, sum of 13 PAHs was detected in the range of 1.3–8.2 ng/g [75]. Similarly, in Mexico, the total amount of detected PAHs did not exceed the permissible limit [76]. The analysis of cow milk samples in California identified PCB-101, PCB-118, PCB-138 as dominant PCBs congeners [69]. However, the the sum of all PCB congeners were below than the permissible tolerance level. Very little amount of PCBs congeners were detected in Slovakia [77]. In another study from UK identified 118, 153, 138 and 180 as dominant PCBs congeners and the total values of all PCBs congeners were in the range of 3.4–16.4 ng/g milk fat [78]. From this analysis, it was also found that some of the milk samples contained higher concentrations of PCBs congeners, which might be due to taking contaminated feeds like contaminated grass by cows.

**Table 7:** The Estimated daily intakes (ng/g), Hazard Risk Index (HI) values of detected PCBs congeners.

**PCBs**	**Mean concentration (ng/kg l.w)**	**EDI**	**ADI**	**HI**
PCB No 52	0.63 ± 0.21	0.2232	40,000 ng/kg	0.00000558
PCB No 101	0.52 ± 0.22	0.184229	40,000 ng/kg	0.00000461
PCB No. 153	2.75 ± 0.41	1.682857	40,000 ng/kg	4.20714E^−05^
PCB No 209	3.35 ± 0.87	1.895429	40,000 ng/kg	4.73857E^−05^
			**Σ HI**	**9.96472E-**^**05**^

Animals are continually exposed to environmental toxins and are able to collect high concentrations of pollutants in their tissues, particularly fat tissues [79]. Therefore, the EU Scientific Committee on Food has fixed values for foods containing several contaminants. [67]. This analysis indicated that approximately 12% of the samples were contaminated with PCBs congeners. However, the concentration of PCBs detected in all milk samples was below the maximum level set by the EU Scientific Committee on Food. A study reported that if the soil is contaminated, it is possible to present PCBs residues in animal-derived foods, including milk, meat, and eggs [80]. While cattle take grass in an open field, they may take up soil contaminated with grass; as a result, contaminants such as PCBs accumulate in their body, and finally those contaminants are transferred to the human body through foods of animal origin.

Studies have reported that amyloidogenesis can be inhibited by polychlorinated biphenyls upon binding to transthyretin in the blood [81]. PCB exposure leads to neurobehavioral changes. PCBs particularly affect the neurotransmitter, dopamine. PCB associated with brominated flame-retardants (BFRs) induces the formation of reactive oxygen species (ROS) in neurons, which are involved in cell death. PCBs and BFRs also have negative effects on the immune system by producing ROS in neutrophils [82]. The endocrine systems of animals and humans can be disrupted by PCBs by controlling natural hormones [83]. PCBs congeners can affect mammalian oocyte maturation and embryonic development. PCBs can interfere with microtubules and alter ooplasm compartmentalization. Ovotoxicity occurred after a miscommunication between the germinal and somatic compartments. Coplanar PCBs can regulate gene expression through aryl hydrocarbon receptor [84]. PCBs can accelerate DNA damage, which can lead to breast cancer. Several chronic inflammatory diseases, such as cardiovascular disease, type 2 diabetes, obesity, hepatic disorders, endocrine dysfunction, and neurological deficits, can develop through PCB exposure [85], [86]. Because of their lipophilic nature, PAHs bind to the cell membranes. As a result, structural changes affect normal cell function [87]. PAHs distribution is mainly dependent on fatty acids in conjugation with triacylglycerides, free cholesterol, and phospholipids, and is later distributed through blood capillaries to tissues such as skeletal muscle tissue, adipose tissue, and liver [88], [89]. PAHs exposure can lead to lung cancer and intestinal diseases in smokers and nonsmokers, respectively [27], [90], [91]. PAHs exposure can lead to the development of cancers, including skin, lung, bladder, and gastrointestinal cancers, as well as damage to several organs such as the liver, kidney, and cataracts. Furthermore, the exposure PAHs causes gene mutation, damage of cells as well as cardiopulmonary related mortality [27], [92].

The metabolism of PAHs occurs through the cytochrome P450 peroxidase and aldo-keto reductase pathways. These pathways involve diol-epoxides and radical cations, which disrupt DNA and damage cells by binding to DNA and proteins. As a result, carcinogenic, mutagenic, immunosuppressive, and teratogenic damage occurs and tumors finally develop tumors [93], [94], [95], [96]. Benzo(a)pyrene (BaP) is associated with delayed hatching in zebrafish by altering the structure of the hatching enzyme (ZHE1). BaP toxicity also affects the skeletal regions of zebrafish by enhancing oxidative stress, apoptosis, and delaying embryonic development [97]. BaP can increase the weight of mice through b-adrenergic mediated stimulation of adipose tissues lipolysis [98]. Lower concentrations of chrysene can diminish cell viability in MIO-M1 cells as a result of apoptosis, whereas higher concentrations can lead to necrosis. Chrysene also alters mitochondrial function. Several studies have shown that PAH can form DNA adducts, leading to cell proliferation [99], [100], [101]. Animal cell culture studies have reported chrysene as a mutagenic, carcinogenic, and genotoxic compound [102], [103], [104]. In male mice, it was observed that immune function and CYP450 activity was altered due to the Chrysene activity [105]. Chrysene was also found to be toxic to rat liver epithelial cells and organisms collected from the sea [106], [107]. A study in C. chanos indicated that anthracene and benzo [a] pyrene can increase oxidative stress and are neurotoxic [108]. The number of cultured hamster cells was reduced after treatment with benzo (a)pyrenediones [109]. Almost similar result was observed with Hexachlorocyclohexane (HCH), an insecticide which significantly reduced the hatching and survivability rate of zebra fishes. Besides this effect, morphological deformities were also observed. Moreover, it induced oxidative stress [110].

PAHs are allocated throughout the environment and persist for a longer time [111]. As PAHs are lipophilic compounds they may contaminate accumulate on high fat containing foods like milk. After accumulation in adipose tissue, PAHs may be excreted in milk [48].

Several PAHs compounds exist; however, only 16 PAHs compounds have been studied so far in most of the studies. These results indicate that environmental pollution plays an important role in the contamination of milk by PAHs [112]. There are several ways to contaminate food. PAHs can contaminate vegetables and fruits in contaminated soil. Fish and seafood can be contaminated by polluted oceans. Cattles can take PAHs along with grasses (sometimes soil from grasses) from contaminated soils in industrial areas. Several studies have shown that milk products contain a remarkable amount of contaminants such as PAHs [113]. Different foods can generate variable levels of PAHs depending on several factors, including duration of heat treatment, temperature, types of fuel used during food production, fat content in food, and smoke utilization during food preparation. Among dairy products, the highest concentration of PAHs is generated in smoked cheese because of the smoke used during preparation and the fat content; environmental toxic elements are bio-accumulated in milk and milk products, transferred to the human body, and finally influence human health. Pasteurized milk samples contain lower PAHs than raw and UHT milk samples because of the differences in fat content [87]. Other studies on milk and milk products also showed similar results [58]. In several other studies, naphthalene, acenaphthylene, acenaphthene, fluorene, and benzo (b) fluoranthene were detected in milk samples [33], [51], [58]. In our study, we detected only benzo (a) anthracene and chrysene at lower concentrations than in other studies performed in several countries worldwide. However, further studies are recommended to ensure public health safety.

## 4 Conclusion

This study investigated the presence of PCBs congeners and PAHs compounds in milk samples from several parts of Dhaka, Bangladesh using GC-MS/MS. The presence of PCBs in milk samples indicates the presence of environmental pollution. There are several other sources of PCBs contamination and there is a possibility of food contamination through PCBs congeners. The majority of PAHs compounds were not detected in milk samples. Very low amounts of benzo (a) anthracene and chrysene were detected in samples from industrial areas, where more human activities were observed. Therefore, proper detection and monitoring are required to ensure the safety of foods from animal sources. Detected values below the recommended levels indicate that the milk consumed by the population is free from toxicological risks. However, long-term exposure may result in significant exposure and cause a high contamination risk. Therefore, further studies are needed to establish specific MRL values for the Bangladeshi population.

## CRediT authorship contribution statement

**G.M.M. Anwarul Hasan:** Conceptualization, Investigation, Methodology, Data curation, Formal analysis, Writing − original draft. **Md. Aftab Ali Shaikh:** Conceptualization, Funding acquisition, Project administration, Writing − review & editing. **Mohammed A. Satter:** Data curation, Formal analysis, Funding acquisition, Investigation, Resources, Software, Supervision, Validation. **Md. Sabir Hossain:** Methodology, Software, Supervision, Validation.

## Declaration of Competing Interest

The authors declare that they have no known competing financial interests or personal relationships that could have appeared to influence the work reported in this paper.
